# Impact of Combined Modulation of Two Potassium Ion Currents on Spiral Waves and Turbulent States in the Heart

**DOI:** 10.3390/e26060446

**Published:** 2024-05-26

**Authors:** Jing Bai, Chunfu Zhang, Yanchun Liang, Adriano Tavares, Lidong Wang

**Affiliations:** 1 School of Statistics and Data Science, Zhuhai College of Science and Technology, Zhuhai 519041, China; baijing@zcst.edu.cn (J.B.); cfzhang@zcst.edu.cn (C.Z.); 2 Department of Industrial Electronics, University of Minho, 4800-058 Guimaraes, Portugal; 3 School of Computer Science, Zhuhai College of Science and Technology, Zhuhai 519041, China

**Keywords:** spiral wave, turbulent states, potassium ion current, control

## Abstract

In the realm of cardiac research, the control of spiral waves and turbulent states has been a persistent focus for scholars. Among various avenues of investigation, the modulation of ion currents represents a crucial direction. It has been proved that the methods involving combined control of currents are superior to singular approaches. While previous studies have proposed some combination strategies, further reinforcement and supplementation are required, particularly in the context of controlling arrhythmias through the combined regulation of two potassium ion currents. This study employs the Luo–Rudy phase I cardiac model, modulating the maximum conductance of the time-dependent potassium current and the time-independent potassium current, to investigate the effects of this combined modulation on spiral waves and turbulent states. Numerical simulation results indicate that, compared to modulating a single current, combining reductions in the conductance of two potassium ion currents can rapidly control spiral waves and turbulent states in a short duration. This implies that employing blockers for both potassium ion currents concurrently represents a more efficient control strategy. The control outcomes of this study represent a novel and effective combination for antiarrhythmic interventions, offering potential avenues for new antiarrhythmic drug targets.

## 1. Introduction

Spiral waves are patches that form through the self-organization of a system when it moves away from equilibrium. They are a specific type of patch commonly observed in excitable media [1,2]. The heart, being a complex and non-uniform excitable system, normally relies on the sinoatrial node to produce regular electrical signals that spread throughout the heart. However, in cases of cardiac damage or dysfunction, irregular electrical signals, known as spiral waves, can appear in the affected cardiac tissue. The presence of spiral waves in the heart has been extensively documented in various studies [3,4,5]. It is well-established that these spiral wave patterns can lead to arrhythmias, posing a significant risk to cardiac function. Spiral waves are traveling waves that self-organize around central defects. When certain parameters are met, these waves can break due to their inherent dynamical instabilities. Initially, new defects appear near the tips of the spiral waves. These new defects then organize themselves to create additional spiral waves, which subsequently generate new defects at their tips. Over time, the number of defects in the system grows until reaching saturation, causing the system to shift into a state of spiral wave turbulence. The transition of spiral waves into turbulent states can lead to ventricular fibrillation, a dangerous condition with significant implications for human health [6,7,8]. As a result, the various dynamic behaviors of spiral waves, especially the control of spiral waves and turbulent states, have been a central focus in research. Computer simulation is a widely used research methodology that has led to the proposal of various control methods [9,10,11,12,13]. These include light control methods [14,15], the polarized electric fields control method [9,16], the far field pacing control method [17,18], the control method for globally applying pulse disturbances [19], the method of applying motion controllers [20], the spiral wave de-nailing control method [21], the control method for local cooling [22], and control methods for regulating cellular ionic currents [23,24,25]. These methods have shown efficiency in controlling spiral waves and turbulent states. However, their clinical application remains a challenge due to the complexity of the heart’s structure and the difficulties in implementation.

There are three common clinical treatments for spiral waves and turbulent states in diseased hearts. In cases of ventricular fibrillation, where spiral waves in the heart break into turbulent states, electrical defibrillation is a frequently used method for emergency interventions [26]. This procedure involves the simultaneous defibrillation of the entire heart using high voltages and energy, but it can lead to severe pain, myocardial dysfunction, and other potential side effects [27]. When arrhythmias are caused by specific structural anomalies, like localized spiral waves in the heart, radiofrequency ablation may be used [28]. This technique utilizes radiofrequency currents to eliminate the structural origins of the spiral waves and treat arrhythmias, but it has drawbacks such as a high recurrence rate and potential complications [29,30]. For most arrhythmias, including stable or meandering spiral waves in the heart, the clinical approach often includes the administration of antiarrhythmic drugs.

Antiarrhythmic drugs function by modulating sodium, calcium, and potassium ion channels in myocardial cells. Through interactions with these specific ion channels, these drugs impact the depolarization and repolarization processes of myocardial cells, as well as the functionality of the conduction system. This modulation is aimed at returning the heart to a normal rhythm, with drug therapy being the most common treatment approach [31]. Different antiarrhythmic drugs target different ion channels, such as those affecting sodium ion channels like ajmaline and flecainide, potassium ion channels like amiodarone and sotalol, calcium ion channels like verapamil and diltiazem, and drugs that impact multiple ion channels like amiodarone and dronedarone [32,33]. The selection of the appropriate antiarrhythmic drugs for treatment is typically based on the mechanisms of the specific arrhythmia. Research has shown that the prolonged use of antiarrhythmic drugs may disturb the ion balance in myocardial tissue, potentially causing arrhythmias or fibrillation [34]. Therefore, the imperative need for the development of more effective and safer novel antiarrhythmic drugs has become apparent, with a focus on identifying improved drug targets gaining considerable attention. Madhvani et al. utilized the dynamic clamp technique to show that targeting the three parameters of L-type calcium currents ($I_{Ca,L}$) effectively inhibits early afterdepolarizations (EADs) and normalizes action potential duration (APD) to reduce repolarization dispersion in isolated rabbit ventricular myocytes. Their findings suggest a promising drug target for developing new antiarrhythmic medications to combat EADs-mediated arrhythmias like polymorphic ventricular tachycardia and ventricular fibrillation. Additionally, they supported their conclusions with computer simulations and proposed that a combination of late sodium current blockers and late L-type calcium current blockers could be a potent antiarrhythmic combination [35,36]. In a separate study, Zhong et al. demonstrated that simultaneously reducing potassium ion conductance and increasing calcium ion conductance in simulations effectively suppressed spiral waves and turbulent states, outperforming the alteration of a single current. As a result, they proposed an innovative antiarrhythmic combination involving the concurrent use of a potassium current blocker and a calcium current enhancer [37].

Potassium ion currents are essential in regulating the excitability and repolarization processes of myocardial cells, exerting a significant influence. Myocardial cells contain multiple types of potassium ion currents, each serving a specific function and duration [38,39]. Grandi et al. utilized a developed mathematical model of human ventricular myocardial cells, which was individually and collectively suppressed by multiple potassium ion currents. They found that these suppressions led to different alterations in APD [38]. Additionally, Landaw et al. demonstrated through multiple simulation models the significant influence of the transient outward potassium current $I_{to}$ in initiating the fragmentation of spiral waves [40]. Therefore, it can be deduced that alterations in various potassium ion currents in different models lead to varying effects on the formation of spiral waves and turbulent states.

Considering that the use of combined ion currents for controlling arrhythmias is superior to relying on a single current, although previous studies have proposed methods for arrhythmia control through combinations of ion currents, there is still a lack of research on the combined modulation of two potassium ion currents for arrhythmia control. Therefore, this paper introduces a strategy involving the simultaneous adjustment of the conductance of two potassium ion currents to control stable spiral waves and turbulent states. The findings of this study offer a new drug target for antiarrhythmic therapy.

Modulating maximum conductance of ion currents is a commonly used technique [37,40]. Therefore, this study adopts the Luo–Rudy (L-R) phase I heart model and adjusts the conductance of two key potassium ion currents in the model [41]: the conductance ${\bar{G}}_{K}$ of the transient outward potassium current $I_{K}$ and the conductance ${\bar{G}}_{K1}$ of the inward rectifier potassium current $I_{K1}$. The research explores the impact of changes in the combination of these two potassium ion currents on the dynamics of spiral waves and turbulent states. The paper provides a parameter range for controlling spiral waves by modulating potassium ion conductance and analyzes the underlying control mechanisms. In our investigation, we found that, compared to the singular reduction of one potassium ion conductance, the simultaneous reduction of both ${\bar{G}}_{K}$ and ${\bar{G}}_{K1}$ provides better control of spiral waves and turbulent states, with a shorter control time. For comparison, we also attempted to simultaneously increase the parameters ${\bar{G}}_{K}$ and ${\bar{G}}_{K1}$. However, the results showed that the approach of simultaneously decreasing ${\bar{G}}_{K}$ and ${\bar{G}}_{K1}$ was more effective in controlling spiral waves and turbulent states, offering a wider range of control. Therefore, based on the control results, we propose that the combined use of inhibitors for both $I_{K}$ and $I_{K1}$ can efficiently control steady-state spiral waves and turbulent states. This represents a novel and effective combination for antiarrhythmic intervention.

The article is organized as follows: Section 2 introduces the mathematical model and control methods used in this study. Section 3 presents the results of numerical simulations, while Section 4 discusses and analyzes the control outcomes. Section 5 provides a summary of the paper.

## 2. Materials and Methods

### 2.1. Mathematical Model

The Luo–Rudy Phase I heart model is a mathematical framework designed by Luo and Rudy to describe alterations in the membrane potential of ventricular cells. This model comprehensively integrates the primary ionic currents present in the cardiac system, providing a more precise representation of the kinetic dynamics of cardiomyocytes. Consequently, it has gained significant traction and is widely employed in numerical simulation research. In our investigation, we have harnessed the capabilities of the L-R Phase I heart model to establish a two-dimensional myocardial tissue construct that encompasses six distinct ionic currents. The model’s kinetic equations are integral to understanding its functionality and are detailed below [41]:

(1) $$\frac{\partial V}{\partial t}=-\frac{I_{ion}}{C_{m}}+D\nabla^{2}V,$$

(2) $$I_{ion}=I_{Na}+I_{Si}+I_{K}+I_{K1}+I_{Kp}+I_{b}.$$

where V is the myocardial cell membrane voltage (in mV), t is the time (in ms), $C_{m}=1.0\;\mu F/{cm}^{2}$ is the membrane capacitance of the cardiomyocyte, $D=0.001\;{cm}^{2}/ms$ is the diffusion coefficient, and $I_{ion}$ is the sum of all transmembrane ionic currents (in $\mu A/{cm}^{2}$). The meaning and expression of each ionic current is given in Table 1. In Table 1, m, h, j, d, f, and x are the gate variables for each ion current; $x_{i}$, ${K1}_{\infty }$, and $K_{p}$ are the functions of the membrane voltages; and $\bar{G}$ and E are the maximum conductivity and energetic equilibrium potential corresponding to each ion current, respectively. The expressions and values of the individual parameters are in accordance with the literature [41], except for special notes.

### 2.2. Numerical Methods

In numerical simulation, the two-dimensional medium of size 8.4 cm×8.4 cm is discretized into 300×300 grid points, and the coordinates of the grid points are denoted by $\left(i,j\right)$. No-flux boundary conditions are used in all of the simulations. The time derivative is obtained using the first-order Euler forward difference method, and the second-order spatial derivative is obtained using the central difference method with a fixed time step of ∆t=0.02 ms and a spatial step of ∆x=∆y=0.028 cm.

### 2.3. Initial State of the System

**Figure 1:**
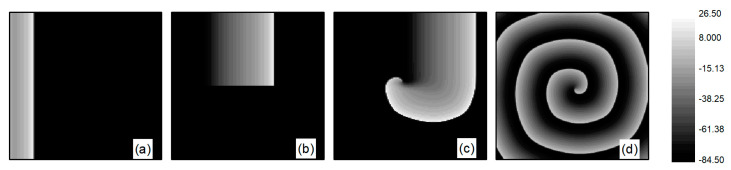
Formation of spiral wave initial state at ${\bar{G}}_{Si}=0.02\;mS/{cm}^{2}$. (**a**) t=20 ms; (**b**) t=100 ms; (**c**) t=140 ms; and (**d**) t=3000 ms.

First, spiral wave and turbulent state primaries are generated in the system. At the initial moment, the system is in the resting state, with the potassium ion maximum conductance parameters fixed at ${\bar{G}}_{K}=0.705\;mS/{cm}^{2}$ and ${\bar{G}}_{K1}=0.6047\;mS/{cm}^{2}$, while ${\bar{G}}_{Si}$ is an adjustable parameter. When conducting spiral wave control studies, ${\bar{G}}_{Si}=0.02\;mS/{cm}^{2}$. Initially, spiral waves are generated under these parameters using the truncated plane wave method, serving as the initial state for spiral wave control studies. Specifically, an external stimulus exceeding the threshold value is applied in a region of 10×300 at the left boundary of the system, setting the voltage in this area to 20 mV for a duration of 0.2 ms. This results in the generation of a train of travelling waves propagating to the right in this region, as depicted in Figure 1a. As the travelling wave propagates to the right to the central position of the system, the travelling wave is truncated and the lower half is removed, i.e., the state variable for the lower half of the system is set to the value of the resting state. This is equivalent to truncating the travelling wave from the middle position. With the evolution of time, the region in the vicinity of the travelling wave truncation will gradually curl and eventually transform into a single spiral wave within the system, as illustrated in Figure 1b–d. The spiral wave shown in Figure 1d is a stable spiral wave after the system has evolved for 3 s, and we use it as the initial state for studying spiral wave control.

**Figure 2:**
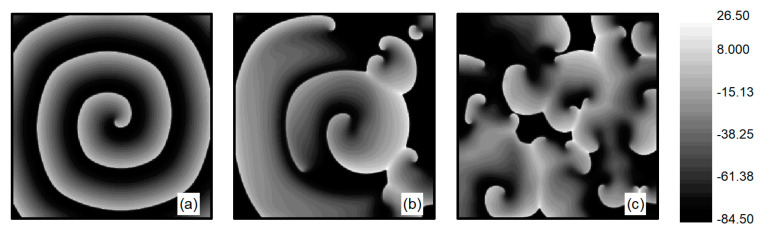
Formation of turbulent states’ initial state at ${\bar{G}}_{Si}=0.05\;mS/{cm}^{2}$. (**a**) t=0 ms; (**b**) t=314 ms; (**c**) t=6000 ms.

The L-type calcium current $I_{Ca,L}$ plays a crucial role in the stability of spiral waves. Adjusting the L-type calcium conductance can alter the slope of the APD restitution curve, thereby influencing spiral wave stability [35]. Therefore, when conducting research on turbulent states control, ${\bar{G}}_{Si}$ is set to $0.05\;mS/{cm}^{2}$. The initial state for turbulent states is generated as follows: employing the single spiral wave depicted in Figure 1d as the initial condition and adjusting the parameter ${\bar{G}}_{Si}=0.05\;mS/{cm}^{2}$, spiral waves within the system undergo fragmentation due to Doppler instability [42]. This process leads to the evolution into turbulent state, as illustrated in Figure 2a–c. The characteristic of turbulent states is that there are many small spiral waves in the system, and they meander randomly. These small spiral waves can disappear and reappear. Turbulent states can potentially be a state of spatiotemporal chaos. Specifically, Figure 2c represents the membrane potential patterns of the system after 6 s of evolution, which we utilize as the initial state for investigating turbulent state control.

Several indicators have been introduced to characterize the dynamical behavior of chaotic systems, including Lyapunov exponents, power spectrum analysis, Poincare section, and autocorrelation function [43,44]. Among these, the Poincare section provides a particularly intuitive description of chaotic motion. The Poincare section is obtained as follows: First, a surface in phase space is selected, known as the Poincare section. Whenever the system’s trajectory crosses this surface in the same direction, the intersection points of the trajectory with the surface are recorded. The distribution of these points is then analyzed to determine the state of the system. If there are a finite number of discrete points on the section, the system is periodic; if the distribution of points on the section forms short lines, the system is quasi-periodic; if the points on the section are distributed irregularly, forming continuous long lines, this indicates that a deterministic system is exhibiting apparently random phenomena, which is characteristic of a chaotic state.

**Figure 3:**
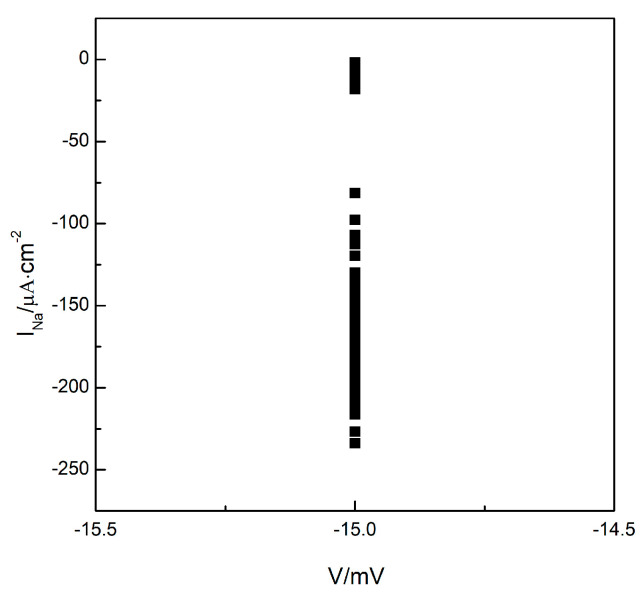
The Poincare section.

To understand whether the turbulent state in the system is a state of spatiotemporal chaos, we plotted the Poincare section as depicted in Figure 3. Maintaining the parameter ${\bar{G}}_{Si}=0.05\;mS/{cm}^{2}$, we allowed the system to evolve further based on the observations in Figure 2c. We observe the changes in membrane potential and sodium ion current at any arbitrary grid point within the system (the results for grid point $\left(100,\;200\right)$ are provided below). We select the membrane potential of V=−15 mV as the Poincare section and record the values of the sodium ion current each time the phase space point, consisting of the membrane potential and sodium ion current, crosses the Poincare section in the direction of increasing membrane potential. We observe that the values of the sodium ion current change irregularly, indicating that the intersections of the phase space trajectories with the Poincare section occurred randomly. These intersections ultimately formed a continuous, long line distribution, as illustrated in Figure 3, indicating that the turbulent states in the system could potentially be states of spatiotemporal chaos.

### 2.4. Control Methods

**Figure 4:**
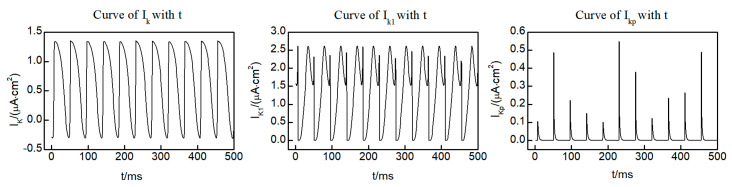
Time dependent curves of three potassium ion currents $I_{K}$, $I_{K1}$, and $I_{Kp}$.

Within the L-R Phase I heart model, three distinct potassium ion currents are present: the time-dependent potassium current $I_{K}$, the time-independent potassium current $I_{K1}$, and the plateau potassium current $I_{Kp}$. Figure 4 shows the curves of the three potassium ion currents with time at the (250, 250) lattice point within the system, taken from the initial state of the spiral wave. Notably, $I_{K}$ and $I_{K1}$ exhibit oscillatory variations over time, while $I_{Kp}$ displays pulsed fluctuations. It is worth mentioning that, except for brief intervals when current values are observed, the majority of the time $I_{Kp}$ remains equal to 0. This observation indicates that $I_{K}$ and $I_{K1}$ exert a more significant influence on the dynamics of the spiral wave. Consequently, our focus for control is directed towards $I_{K}$ and $I_{K1}$.

We vary the maximum conductivity ${\bar{G}}_{K}$ and ${\bar{G}}_{K1}$ of $I_{K}$ and $I_{K1}$ to study the variation of spiral waves and turbulent states. Therefore, by setting ${\bar{G}}_{K}=\lambda_{K}\cdot 0.705\;mS/{cm}^{2}$ and ${\bar{G}}_{K1}=\lambda_{K1}\cdot 0.6047\;mS/{cm}^{2}$ and using the reduction of maximum potassium conductance to control spiral waves and turbulent states, the values for $\lambda_{K}$ and $\lambda_{K1}$ range from $\left[0.1,\;1\right]$ with an increment of 0.1. When using the increase in maximum potassium conductance to control spiral waves and turbulent states, the values for $\lambda_{K}$ and $\lambda_{K1}$ range from $\left[1,\;10\right]$ with an increment of 1. We study the variation of spiral waves and turbulent states as the parameters $\lambda_{K}$ and $\lambda_{K1}$ are varied in both methods. Since simply decreasing or increasing the maximum conductance of potassium ion currents does not result in new fixed points in the equations, there are no abrupt changes in the system’s dynamical behavior. Consequently, within the specified ranges of changes for parameters $\lambda_{K}$ and $\lambda_{K1}$, the various phenomena arising from reducing and increasing the maximum conductance of potassium ion currents can be observed. This allows for a more effective evaluation of the control effects.

### 2.5. Measurement of Control Effects

We use the changes in the average membrane potential difference $\bar{V}$ to measure the control effectiveness of spiral waves and turbulent states [20,23], which is given by:

(3) $$\bar{V}=\frac{1}{300^{2}}\sum_{i,j=1}^{300}\left|V-V_{r}\right|.$$

where $V_{r}=-83.0\;mV$ is the value of resting membrane potential of myocardial cells. The excitation of cardiac myocytes can be determined by analyzing their membrane potentials. By calculating the difference between the membrane potentials of all cardiac myocytes and their resting potentials, it is possible to identify excited myocytes in the system. In the spiral wave state, the variable $\bar{V}$ shows regular oscillations over time, whereas in turbulent states, $\bar{V}$ oscillates irregularly. When spiral waves or turbulent states disappear, all cardiac myocytes return to their resting state, with $\bar{V}$ stabilizing over time. If cells are not hyperpolarized due to control measures, $\bar{V}$ tends towards zero; otherwise, it approaches a small constant value. The time taken for $\bar{V}$ to reach a small constant value from the start of control is known as the control time, with shorter control times indicating more effective control.

## 3. Results

To examine the impact of potassium ion current regulation on spiral waves and turbulent states, we initiated control on the system from the initial state at t=0 ms, simultaneously decreasing or increasing the control parameters $\lambda_{K}$ and $\lambda_{K1}$. If spiral waves and turbulent states can be controlled within 3 s, this indicates that the control is effective. The numerical simulation results show that adjusting the potassium ion current can make the spiral wave diffuse and can control the spiral wave and turbulent states in the system. This investigation reveals the existence of a controllable range wherein the control mechanisms are effective.

### 3.1. The Control Effects on the System at the Initial State of Spiral Waves

**Figure 5:**
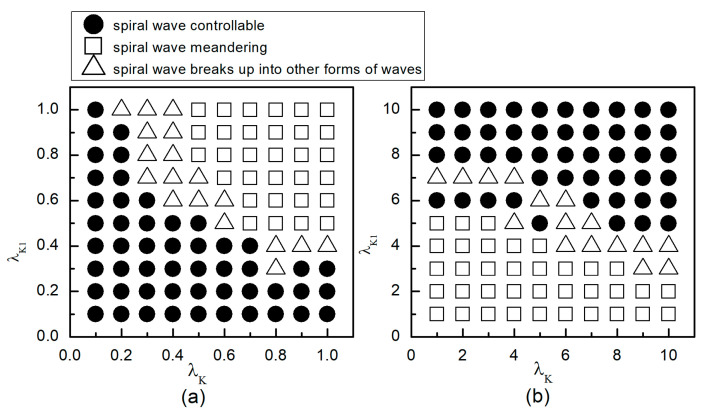
Phase diagram in the $\lambda_{K}-\lambda_{K1}$ parameter plane. (**a**) the variation range of $\lambda_{K}$ and $\lambda_{K1}$ is $\left[0.1,\;1\right]$; (**b**) the variation range of $\lambda_{K}$ and $\lambda_{K1}$ is $\left[1,\;10\right]$.

When the system is initially in a spiral wave state, applying different control parameters $\lambda_{K}$ and $\lambda_{K1}$ results in varied changes in the spiral waves; the greater the change in control parameters, the larger the impact on the spiral waves. To more directly understand the changes in spiral waves, Figure 5 depicts the phase diagram in the $\lambda_{K}-\lambda_{K1}$ parameter plane. Notably, modifying conductivity by either increasing $\lambda_{K}$, $\lambda_{K1}$ or decreasing $\lambda_{K}$, $\lambda_{K1}$ yields several significant changes in the spiral wave dynamics. These changes can be categorized into three primary outcomes: the first type of change is indicated by solid symbols ● in Figure 5, where the parameters corresponding to these symbols result in the spiral waves being controlled and eliminated from the system. The second type of change, represented by hollow symbols ∆ in Figure 5, occurs under parameters where the spiral waves are not controlled; these waves fracture or fragment, subsequently evolving into multiple spiral waves, spiral wave pairs, or a single spiral wave. The third type of change, denoted by hollow symbols □ in Figure 5, occurs under parameters where the spiral waves neither break nor fragment but rather continue to meander within the system as spiral waves.

As can be observed from Figure 5a: (1) With the decrease in the values of $\lambda_{K}$ or $\lambda_{K1}$, local conduction blocks occur, leading to the fragmentation of spiral waves, which evolve into single or multiple spiral waves; (2) Further reduction in the values of $\lambda_{K}$ or $\lambda_{K1}$ results in the control of spiral waves. By analyzing the controllable region of spiral waves depicted in Figure 5a, it is observed that reducing $\lambda_{K1}$ to below 0.4 allows for the control of spiral waves without substantial changes to $\lambda_{K}$. Conversely, if the value of $\lambda_{K1}$ is high, a significantly reduced value of $\lambda_{K}$ is necessary to achieve control of the spiral waves.

From Figure 5b, it can be observed that: (1) When the value of $\lambda_{K1}$ is small, increasing $\lambda_{K}$ does not alter the meandering state of the spiral waves, nor does enable their control; (2) When $\lambda_{K}$ is low, a significantly large value of $\lambda_{K1}$ is required to control the spiral waves, resulting in a direct transition from meandering state to controllable state, which is different from scenarios where both $\lambda_{K}$ and $\lambda_{K1}$ are reduced; (3) When $\lambda_{K1}$ is increased to 8, the spiral waves can be controlled without altering $\lambda_{K}$; and (4) Increasing both $\lambda_{K}$ and $\lambda_{K1}$ simultaneously expands the control range of the spiral waves.

Therefore, combining the observations from Figure 5a,b, we find that adjusting $\lambda_{K1}$ can control spiral waves, but this usually requires a substantial modification of $\lambda_{K1}$. If $\lambda_{K}$ and $\lambda_{K1}$ are adjusted concurrently, appropriately decreasing or increasing both parameters can achieve the same effect in controlling the spiral waves. Next, we will analyze the mechanisms of spiral wave control when reducing and increasing $\lambda_{K}$ and $\lambda_{K1}$, respectively.

#### 3.1.1. The Effect of Reducing the Maximum Conductance of Potassium Ions on Spiral Waves

**Figure 6:**
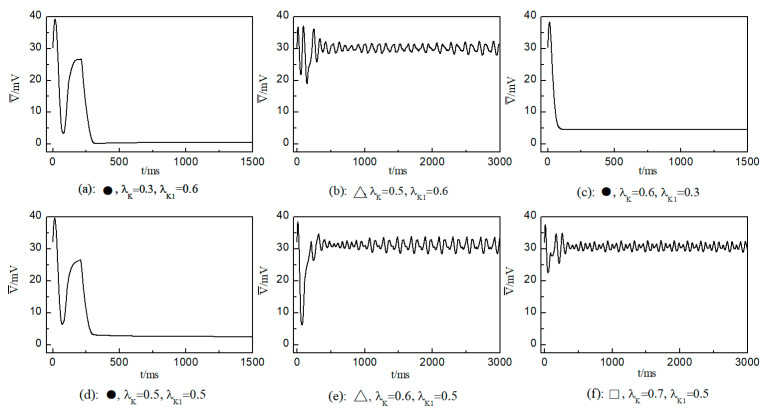
Curve of average membrane potential difference $\bar{V}$ with time t for different control parameters; the symbols ●, ∆, and □ in the figure correspond to the symbols in Figure 5a.

We first consider the effect on the spiral wave when both $\lambda_{K}$ and $\lambda_{K1}$ are varied in the range $\left[0.1,\;1\right]$. Figure 6 portrays the time-dependent variation curve of the average membrane potential difference under different parameters. This change curve provides insights into the control status of spiral waves in the system.

**Figure 7:**
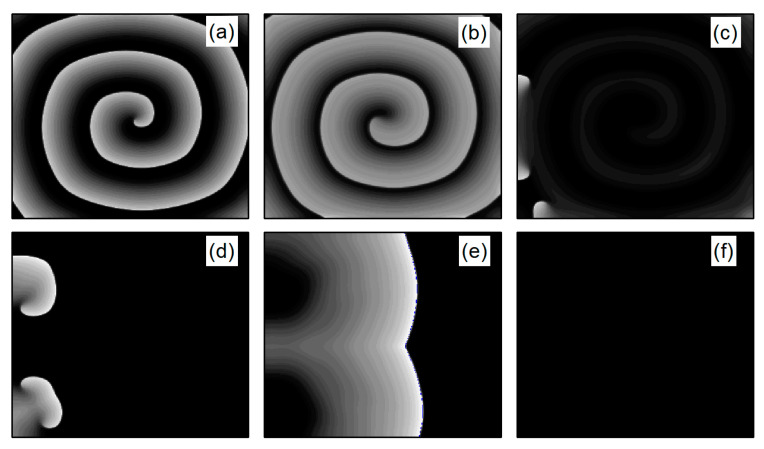
Pattern of the membrane potential at different time moments for $\lambda_{K}=0.3$, $\lambda_{K1}=0.6$, corresponding to the parameters in Figure 6a. (**a**) t=0 ms; (**b**) t=22 ms; (**c**) t=62 ms; (**d**) t=94 ms; (**e**) t=164 ms; and (**f**) t=334 ms.

In the case of Figure 6a, the average membrane potential difference $\bar{V}$ rapidly approaches zero after two oscillations. To understand the control mechanisms involved, Figure 7 presents membrane potential patterns at different time points under the parameters of Figure 6a. From Figure 7, it is evident that after the application of control, the decrease in parameters ${\bar{G}}_{K}$ and ${\bar{G}}_{K1}$ leads to the reduction in $I_{K}$ and $I_{K1}$, resulting in a slowed repolarization process in cardiac myocytes and an extended APD (the thickening of the spiral wave arms), as shown in Figure 7b. This leads to conduction disturbances, causing most of the spiral waves to disappear, with only three small waves remaining at the boundaries (see Figure 7c). These evolve into spiral waves (see Figure 7d), which is the cause of the second peak observed in Figure 6a. Once the spiral wave fronts meander beyond the boundary, the spiral waves disappear, indicating that the spiral waves are controlled.

As shown in Figure 6c, the spiral waves are controlled, and the control time is shorter. This is attributed to the longer APD of cardiac myocytes when values of $\lambda_{K}$ and ${\;\lambda }_{K1}$ are relatively low, allowing the spiral waves to disappear directly through conduction disturbances, thus shortening the control time. From Figure 6a,c, it is evident that within an appropriate range of parameter variations, controlling both ${\bar{G}}_{K}$ and ${\bar{G}}_{K1}$ simultaneously allows spiral waves to disappear quickly. Furthermore, after ceasing control, the dynamics of the heart remain unaffected.

**Figure 8:**
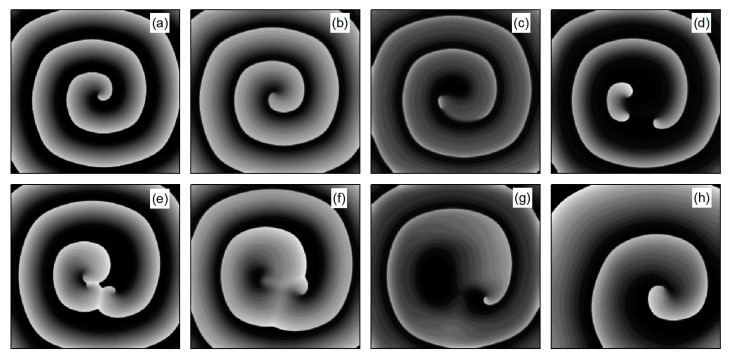
Pattern of the membrane potential at different time moments for $\lambda_{K}=0.5$, $\lambda_{K1}=0.6$, corresponding to the parameters in Figure 6b. (**a**) t=0 ms; (**b**) t=16 ms; (**c**) t=42 ms; (**d**) t=62 ms; (**e**) t=80 ms; (**f**) t=96 ms; (**g**) t=126 ms; and (**h**) t=356 ms.

As depicted in Figure 6b, $\bar{V}$ stabilizes within a narrow range after several oscillations. To understand the underlying physical mechanisms, Figure 8 provides membrane potential patterns at different times during the control process. From Figure 8, it is apparent that control induces localized conduction disturbances, resulting in partial fracturing of the spiral waves. At the fracture sites, pairs of spiral waves form (see Figure 8e). During this process, the waves within the system are unstable, leading to oscillations in the average membrane potential difference $\bar{V}$. As time progresses, the two wave fronts collide and then disappear, ultimately leaving only a single spiral wave in the system. Consequently, $\bar{V}$ fluctuates within a narrow range.

**Figure 9:**
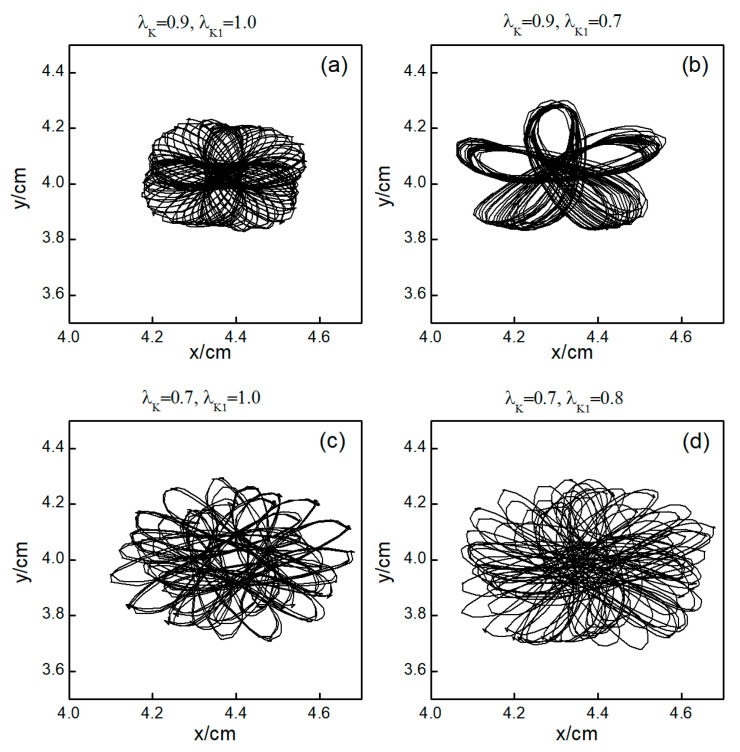
Trajectories of spiral wave tip for different control parameters. (**a**) $\lambda_{K}=0.9$, $\lambda_{K1}=1.0$; (**b**) $\lambda_{K}=0.9$, $\lambda_{K1}=0.7$; (**c**) $\lambda_{K}=0.7$, $\lambda_{K1}=1.0$; and (**d**) $\lambda_{K}=0.7$, $\lambda_{K1}=0.8$.

The control mechanisms in Figure 6d,e are the same as those in Figure 6a,b, respectively. In Figure 6f, $\bar{V}$ stabilizes within a small range after several minor oscillations. In this scenario, the spiral waves did not fracture; instead, different degrees of meandering occurred in the wave tips following the application of control. For the uninterrupted spiral wave, in order to observe their motion, we used the intersection of two contours with a cell membrane potential of −35 mV to determine the tip of the spiral wave, and the interval between the two contours was 2 ms [23]. Figure 9 shows the tip trajectory of the spiral wave under different parameters, corresponding to the hollow symbol □ in Figure 5a. We found that when parameters $\lambda_{K}$ and $\lambda_{K1}$ were not reduced much, although they did not cause the spiral wave to break or disappear, the change in parameters still affected the movement of the spiral wave. This suggests that alterations in potassium ion currents modify the shape of the spiral wavefront trajectories. Reducing parameters $\lambda_{K}$ and $\lambda_{K1}$ increases the meandering amplitude of the spiral waves, impacting their stability and causing the wavefronts’ meandering trajectories to transition from regular to irregular patterns.

#### 3.1.2. The Effect of Increasing the Maximum Conductance of Potassium Ions on Spiral Waves

**Figure 10:**
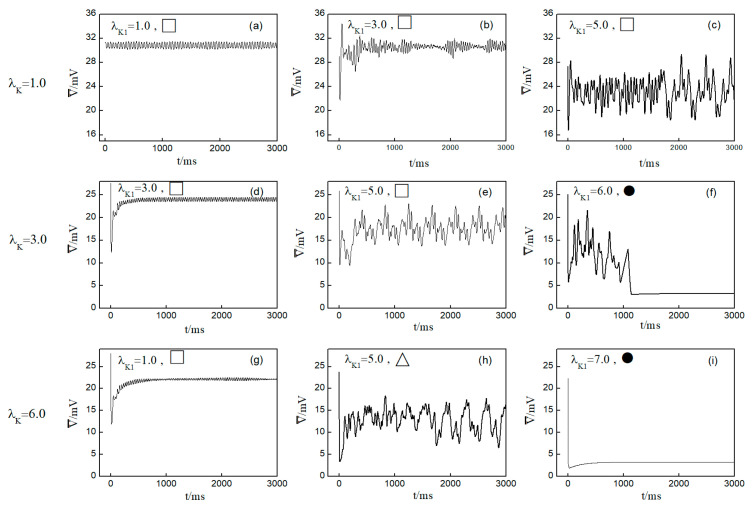
Curve of average membrane potential difference $\bar{V}$ with time t for different control parameters; the symbols ●, ∆, □ in the figure correspond to the symbols in Figure 5b. (**a**) $\lambda_{K}=1.0$, $\lambda_{K1}=1.0$; (**b**) $\lambda_{K}=1.0$, $\lambda_{K1}=3.0$; (**c**) $\lambda_{K}=1.0$, $\lambda_{K1}=5.0$; (**d**) $\lambda_{K}=3.0$, $\lambda_{K1}=3.0$; (**e**) $\lambda_{K}=3.0$, $\lambda_{K1}=5.0$; (**f**) $\lambda_{K}=3.0$, $\lambda_{K1}=6.0$; (**g**) $\lambda_{K}=6.0$, $\lambda_{K1}=1.0$; (**h**) $\lambda_{K}=6.0$, $\lambda_{K1}=5.0$; and (**i**) $\lambda_{K}=6.0$, $\lambda_{K1}=7.0$.

**Figure 11:**
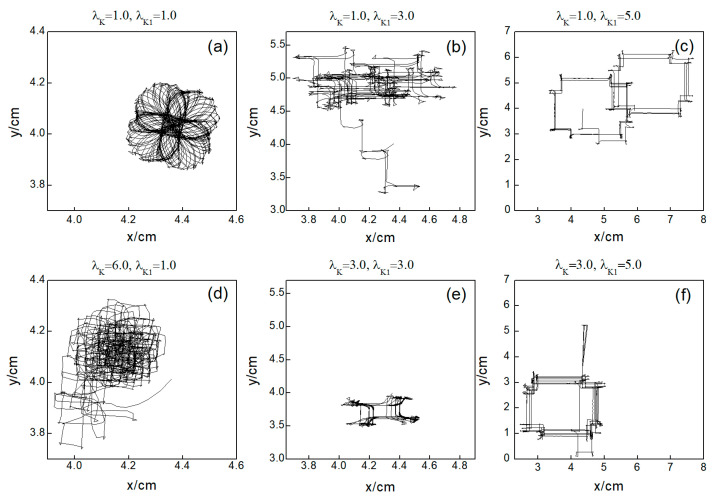
Trajectories of spiral wave tip for different control parameters. (**a**) $\lambda_{K}=1.0$, $\lambda_{K1}=1.0$; (**b**) $\lambda_{K}=1.0$, $\lambda_{K1}=3.0$; (**c**) $\lambda_{K}=1.0$, $\lambda_{K1}=5.0$; (**d**) $\lambda_{K}=6.0$, $\lambda_{K1}=1.0$; (**e**) $\lambda_{K}=3.0$, $\lambda_{K1}=3.0$; and (**f**) $\lambda_{K}=3.0$, $\lambda_{K1}=5.0$.

Next, we elaborate on the effect on spiral waves when both $\lambda_{K}$ and $\lambda_{K1}$ are varied in the range $\left[1,\;10\right]$. Figure 10 shows the changes in the average membrane potential difference under different parameters. Notably, Figure 10a–e,g correspond to the spiral waves that remain unbroken and uncontrolled under the applied parameters. Their corresponding wave tips trajectories are plotted in Figure 11. Figure 10a and Figure 11a were obtained with parameters $\lambda_{K}=1.0$ and $\lambda_{K1}=1.0$, reflecting the absence of control.

Comparing the results presented in Figure 10a–e,g, it can be seen that after applying the control, the value of $\bar{V}$ has a single large change at the beginning of the control. As parameters $\lambda_{K}$ or $\lambda_{K1}$ are increased, $\bar{V}$ begins to exhibit low-amplitude oscillations, which gradually evolve into high-amplitude oscillations as the parameter values increase further. This behavior can be attributed to the increased potassium ion current and the resultant shortening of the action potential duration post-control. This shortening, in turn, reduces the repolarization time of cardiomyocytes, lowering the medium’s excitability and making it less prone to excitation. Consequently, wavefronts within the system exhibit varying degrees of meandering, with larger control parameters leading to more substantial wavefront meandering, as demonstrated in Figure 11.

In Figure 10h, $\bar{V}$ undergoes a transformation from irregular oscillations to regular oscillations after a significant change. This shift is attributable to the diminished excitability of the medium, causing the spiral wave to fragment into many small spiral waves. The wave tips of each small spiral wave continue to move and interact with each other, resulting in the continuous disappearance of small spiral waves while new ones are generated. This process continues until only a single spiral wave remains within the system.

**Figure 12:**
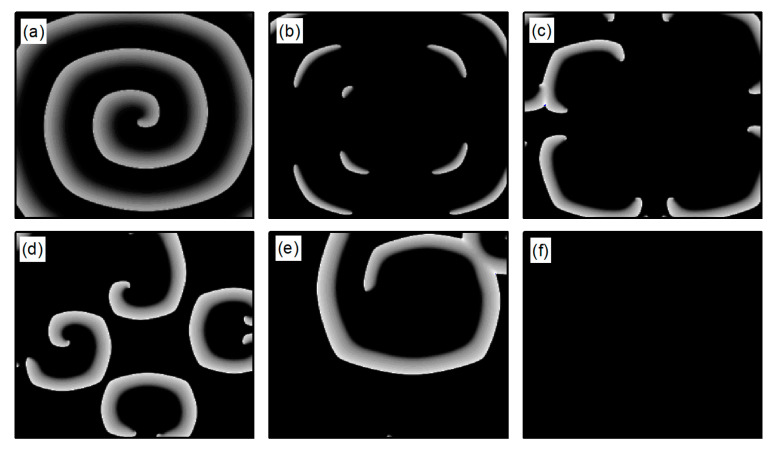
Pattern of the membrane potential at different time moments for $\lambda_{K}=3.0$, $\lambda_{K1}=6.0$, corresponding to the parameters in Figure 10f. (**a**) t=2 ms; (**b**) t=20 ms; (**c**) t=70 ms; (**d**) t=180 ms; (**e**) t=560 ms; and (**f**) t=1160 ms.

From Figure 10f,i, it is evident that following the control of spiral waves, $\bar{V}$ stabilizes at a positive value close to zero. This occurs because an increase in potassium ion current leads to hyperpolarization of the cardiac myocytes. During hyperpolarization, the membrane potential is lower than the resting potential $V_{r}=-83.0\;mV$, resulting in $\bar{V}$ not being zero. Comparing Figure 10f,i, it is evident that $\bar{V}$ changes over time differently in each case. Figure 10f shows that $\bar{V}$ oscillates over time before transitioning to a constant. This is because control allows the cells to return to a resting state and then enter a state of hyperpolarization, which greatly reduces cell excitability. This leads to the fracturing of the spiral wave arms, and the spiral wave breaks into multiple spiral waves. At this point, $\bar{V}$ oscillates as the multiple spiral waves interact and evolve into a single spiral wave. When this single spiral wave meanders out of the system, the spiral waves disappear, and $\bar{V}$ transitions to a constant, as illustrated in Figure 12. This mode of disappearance corresponds to the isolated five solid symbols ● in Figure 5b.

**Figure 13:**
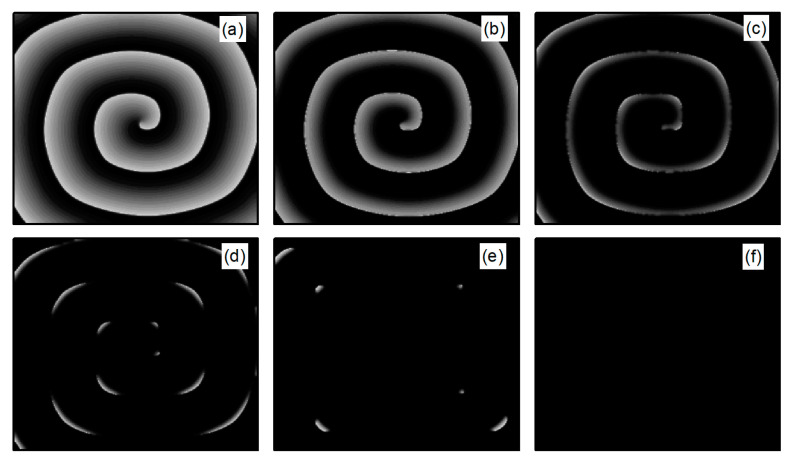
Pattern of the membrane potential at different time moments for $\lambda_{K}=6.0$, $\lambda_{K1}=7.0$, corresponding to the parameters in Figure 10i. (**a**) t=0.02 ms; (**b**) t=2 ms; (**c**) t=4 ms; (**d**) t=6 ms; (**e**) t=16 ms; and (**f**) t=30 ms.

Figure 10i shows that $\bar{V}$ directly decays to a constant, which occurs because control brings the cells back to a resting state and then into a state of hyperpolarization. The membrane potential is lower than that in Figure 10f, rendering the cells unexcitable, as shown in Figure 13. Consequently, the spiral waves disappear directly, resulting in the shortest control time. The controllable region depicted in Figure 5b primarily corresponds to this scenario.

Through comparing the changes in spiral waves under conditions of both decreased and increased potassium ion maximum conductance, we find that minor alterations in conductance result in varying degrees of spiral wave meandering. Increasing the magnitude of conductance change generally leads to spiral wave fragmentation. Further amplification of conductance change results in the control of the spiral waves. However, the mechanisms of spiral wave control differ under the two conditions. When the maximum conductance of potassium ions is decreased, spiral waves disappear from the system due to conduction blocks, resulting in a shorter control time within the controllable region depicted in Figure 5a. Conversely, when the maximum conductance of potassium ions is increased, the excitability of the system decreases, leading to the control of spiral waves, with the extent of the reduction in system excitability determining the duration of spiral wave control. When myocardial cells are difficult to excite, spiral waves do not disappear directly but fragment into multiple spiral waves before vanishing, as shown in Figure 12. Under these circumstances, the control duration is typically longer. Conversely, when myocardial cells cannot be excited, spiral waves disappear directly, as illustrated in Figure 13. In such cases, the control duration is notably short, and further increases in potassium ion conductance do not alter the control time. Thus, within the controllable region depicted in Figure 5b, control durations under certain parameters are extended.

### 3.2. The Control Effects on the System at the Initial State of Turbulent States

**Figure 14:**
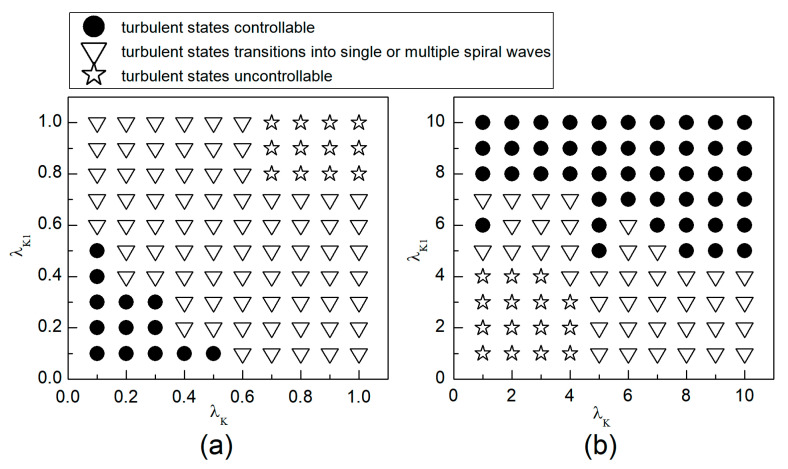
Phase diagram in the $\lambda_{K}-\lambda_{K1}$ parameter plane. (**a**) The variation range of $\lambda_{K}$ and $\lambda_{K1}$ is [0.1, 1]; (**b**) the variation range of $\lambda_{K}$ and $\lambda_{K1}$ is $\left[1,\;10\right]$.

The following study examines the impact of potassium ion current changes on turbulent states. Figure 14 presents the phase diagram on the $\lambda_{K}-\lambda_{K1}$ parameter plane. The diagram illustrates that, by adjusting the control parameters $\lambda_{K}$ and $\lambda_{K1}$ within a specified range, the conversion of turbulent states into either single or multiple spiral waves can be observed. Moreover, the judicious selection of these parameters can result in the turbulent states either transforming into a single spiral wave that drifts and vanishes from the system, or disappearing directly. The control mechanism involved is analogous to that used in spiral wave management.

**Figure 15:**
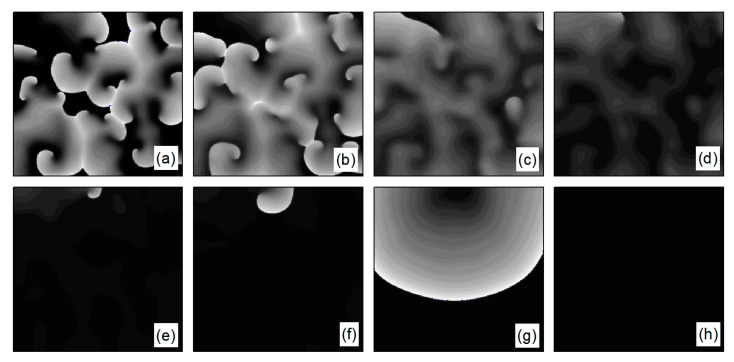
Pattern of the membrane potential at different time moments for $\lambda_{K}=0.3$, $\lambda_{K1}=0.3$ (**a**) t=0 ms; (**b**) t=12 ms; (**c**) t=36 ms; (**d**) t=64 ms; (**e**) t=106 ms; (**f**) t=122 ms; (**g**) t=200 ms; and (**h**) t=460 ms.

We first explore the behavior of turbulent states under control parameters within the $\left[0.1,\;1\right]$ range. A comparison between Figure 5a and Figure 14a reveals a reduced controllable region for turbulent states relative to spiral waves, a reduction due to the inherent instability and complexity of turbulent states, which complicates control efforts. For an intuitive understanding of the control effects on turbulent states, Figure 15 illustrates the changes in membrane potential patterns at different times under conditions of $\lambda_{K}=0.3$ and $\lambda_{K1}=0.3$. As evident from Figure 15, following the application of control, the majority of the turbulent states disappear due to conduction blocks, while the remaining turbulent states at the boundaries evolve into small spiral waves. Once the wavefronts of these spirals meander out of the system, the turbulent states are effectively controlled. Compared with the current control parameters $\lambda_{K}=0.3$ and $\lambda_{K1}=0.3$, if $\lambda_{K}$ or $\lambda_{K1}$ is further reduced, turbulent states will disappear from the system due to conduction blocks. Conversely, if the parameters $\lambda_{K}$ or $\lambda_{K1}$ are increased, the transformation of turbulent states into single or multiple spiral waves can be observed.

**Figure 16:**
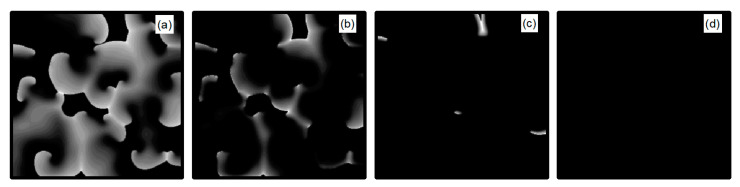
Pattern of the membrane potential at different time moments for $\lambda_{K}=6.0$, $\lambda_{K1}=7.0$ (**a**) t=1 ms; (**b**) t=4 ms; (**c**) t=14 ms; and (**d**) t=140 ms.

Next, we discuss the behavior of turbulent states within the control parameter range of $\left[1,\;10\right]$. Comparing Figure 5b and Figure 14b, we observe that the controllable region for turbulent states remains largely unchanged within this range. This stability can be attributed to the fact that when the control parameters are set relatively high, the APD decreases rapidly, allowing cardiac cells to quickly return to a resting state. Subsequently, the cardiac cells undergo hyperpolarization and become nearly unexcitable. This results in significantly reduced excitability of the system, preventing both spiral waves and turbulent states from propagating within the system, leading to their direct elimination. Figure 16 displays the changes in the membrane potential patterns at different times under conditions of $\lambda_{K}=6.0$ and $\lambda_{K1}=7.0$, illustrating that the turbulent states vanish due to the decreased excitability of the medium.

Compared with the control of spiral waves, we find that the control mechanisms of turbulent states are identical to those of spiral waves. Upon application of control, the parameter range within which turbulent states transitions to multiple or single spiral waves is expanded. Within the controllable region for turbulent states, the duration of control is longer compared to that of spiral waves.

## 4. Discussion

In this paper, we propose a novel approach to suppress spiral waves and turbulent states: the combined modulation of the maximum conductance of two potassium ion currents, $I_{K}$ and $I_{K1}$. Previous research has consistently shown that, compared to the modulation of a single ion current, controlling multiple ion currents simultaneously provides more effective regulation of spiral waves and turbulent states. The findings of this study further substantiate this perspective. From Figure 5a, it can be observed that when $\lambda_{K1}=1.0$, meaning no control is applied to $I_{K1}$, efficient control of spiral waves is achieved only by reducing $\lambda_{K}$ to a very small value. Conversely, when $\lambda_{K}=1.0$, $\lambda_{K1}$ needs to be adjusted to a larger extent for effective control. However, when $\lambda_{K}$ and $\lambda_{K1}$ are both adjusted simultaneously, they only require minor adjustments to achieve optimal control results.

We have discovered that reducing the maximum conductance of $I_{K}$ and $I_{K1}$ simultaneously yields better control results than increasing them. Specifically, effective control of spiral waves and turbulent states can be achieved by reducing the potassium ion maximum conductance by 50% to 70%, whereas an increase in the potassium ion maximum conductance needs to exceed fivefold for similar efficacy. Thus, when changes in the potassium ion maximum conductance are minimal, decreasing $I_{K}$ and $I_{K1}$ simultaneously provides a broader controllable range.

Through numerical simulation studies, we have found: (1) As long as the control parameters are adjusted within the controllable area, it is generally possible to quickly control both spiral waves and turbulent states. The control time for turbulent states is slightly longer compared to spiral waves; (2) The control mechanisms of the two methods are distinct; $I_{K}$ primarily affects the repolarization process of cardiac cells, while $I_{K1}$ mainly functions during the resting state and late repolarization phase. Simultaneously inhibiting these two currents increases the APD, significantly slowing down the repolarization process of cardiac cells. Excited cardiac cells take a long time to return to the resting state, prolonging the effective refractory period of the cardiac cells. This leads to the annihilation of excitation waves in the direction of propagation due to conduction blocks, causing them to extinguish. Conversely, increasing these two currents simultaneously decreases the APD of cardiac cells. Moreover, cardiac cells enter a hyperpolarized state, making them less excitable, significantly reducing the medium’s excitability and resulting in the disappearance of spiral waves. Among these two control mechanisms, inducing conduction blocks to control spiral waves is a commonly employed method. Therefore, the combined use of inhibitors for $I_{K}$ and $I_{K1}$ represents a more effective direction for control.

The control strategy proposed in this article only requires relatively low concentrations of inhibitors for currents $I_{K}$ and $I_{K1}$, appropriately reducing their maximum conductance, to achieve robust control over spiral waves and turbulent states. This approach reduces the side effects resulting from excessive drug use. Based on the results of this study, inhibitors for channels of two potassium ion currents $I_{K}$ and $I_{K1}$ can be considered as a new combination for antiarrhythmic therapy.

## 5. Conclusions

This paper employs the Luo–Rudy Phase I cardiac model to investigate the effects of simultaneously regulating the maximum conductance of $I_{K}$ and $I_{K1}$ on spiral waves and turbulent states. The research findings indicate: (1) Within a certain range, the simultaneous regulation of the maximum conductance of two types of potassium ion currents can rapidly suppress both spiral waves and turbulent states in a short period of time; (2) The control mechanism that involves simultaneously decreasing the maximum conductance of both potassium ion currents is more effective than that involving their increase; (3) Compared to controlling the maximum conductance of a single ion current, the method of combined suppression of the maximum conductance of two types of potassium ion currents offers a broader range of control and shorter control time, indicating superior control efficacy. Therefore, this paper proposes a novel control approach: the combined use of channel blockers for two types of potassium ion currents, $I_{K}$ and $I_{K1}$, to control spiral waves and turbulent states. This method provides valuable insights for the treatment of arrhythmias.

The control effectiveness of this study, although better than that of a single current, and the simultaneous adjustment of potassium ion currents reduce the difficulty of control; further exploration is needed for more efficient methods of controlling spiral waves and turbulent states. Additionally, in future research, we will include a stability analysis of spiral waves and provide a quantitative description of whether the turbulent states are spatiotemporally chaotic.

## Figures and Tables

**Table 1:** Meaning and expression for each ionic current in the L-R model.

Ion Current Expression	Ion Current Meaning
$I_{Na}={\bar{G}}_{Na}\cdot m^{3}\cdot h\cdot j\cdot \left(V-E_{Na}\right)$	the fast inward sodium ion current
$I_{Si}={\bar{G}}_{Si}\cdot d\cdot f\cdot \left(V-E_{Si}\right)$	the slow inward calcium ion current
$I_{K}={\bar{G}}_{K}\cdot x\cdot x_{i}\cdot \left(V-E_{K}\right)$	the time-dependent potassium current
$I_{K1}={\bar{G}}_{K1}\cdot {K1}_{\infty }\cdot \left(V-E_{K1}\right)$	the time-independent potassium current
$I_{Kp}={\bar{G}}_{Kp}\cdot K_{p}\cdot \left(V-E_{Kp}\right)$	the plateau potassium current
$I_{b}={\bar{G}}_{b}\cdot \left(V-E_{b}\right)$	the background leakage current

## Data Availability

The raw data supporting the conclusions of this article will be made available by the authors on request.
